# Polyphenol-Based Prevention and Treatment of Cancer Through Epigenetic and Combinatorial Mechanisms

**DOI:** 10.3390/nu17040616

**Published:** 2025-02-08

**Authors:** Neha Singaravelan, Trygve O. Tollefsbol

**Affiliations:** 1 Department of Biology, University of Alabama at Birmingham, Birmingham, AL 35294, USA; nsingara@uab.edu; 2 Integrative Center for Aging Research, University of Alabama at Birmingham, Birmingham, AL 35294, USA; 3 O’Neal Comprehensive Cancer Research, University of Alabama at Birmingham, Birmingham, AL 35294, USA; 4 Nutrition Obesity Research Center, University of Alabama at Birmingham, Birmingham, AL 35294, USA; 5 Comprehensive Diabetes Center, University of Alabama at Birmingham, Birmingham, AL 35294, USA

**Keywords:** epigenetics, dietary polyphenols, combination, cancer

## Abstract

Polyphenols have been shown to be utilized as an effective treatment for cancer by acting as a DNMT or HDAC inhibitor, reducing inflammatory processes, and causing cell cycle arrest. While there have been many studies demonstrating the anti-cancerous potential of individual polyphenols, there are limited studies on the combinatorial effects of polyphenols. This review focuses on how combinations of different polyphenols can be used as a chemotherapeutic treatment option for patients. Specifically, we examine the combinatorial effects of three commonly used polyphenols: curcumin, resveratrol, and epigallocatechin gallate. These combinations have been shown to induce apoptosis, prevent colony formation and migration, increase tumor suppression, reduce cell viability and angiogenesis, and create several epigenetic modifications. In addition, these anti-cancerous effects were synergistic and additive. Thus, these findings suggest that using different combinations of polyphenols at the appropriate concentrations can be used as a better and more efficacious treatment against cancer as compared to using polyphenols individually.

## 1. Introduction

Cancer is defined as the abnormal growth of cells. This life-threatening disease can stem from any organ or area of the body [1]. As of 2024, there have been 2,001,140 new cases and 611,720 people have died from the disease. The most common types of cancer are breast cancer, prostate cancer, colon and rectum cancer, and skin cancer [2]. Current standards of treatment for cancer provide numerous benefits and can significantly inhibit the progression of the disease; however, it also has considerable drawbacks, such as increasing the risk of developing secondary diseases, inducing immunosuppression, and being difficult to develop and efficiently deliver in the body. In addition, while the biological side effects of treatments are often highlighted, the sociological effects are ignored. These treatments are very expensive, presenting an obstacle to those from low socioeconomic backgrounds. Furthermore, many areas of the world have a lack of access to cancer treatments, as they often do not have the technology and equipment to develop these drugs and therapies. This creates an issue of inequality between those who have an increased advantage due to their location or financial background compared to those who are from smaller, more rural areas. Thus, this presents the need to explore other avenues for cancer treatment that are more accessible, affordable, present less side effects, and can be used as a preventive method for cancer treatment. According to the American Institute for Cancer Research, 30–40% of all cancers can be prevented by appropriate diets. Specific nutrients can have an impact on tumor formation and reduce cancer risk [3]. Polyphenols can block cell cycle progression, induce apoptosis, and attack tumor formation while also having limited toxic side effects. More importantly, these molecules are more accessible as they are found in everyday food items, allowing them to be utilized as a preventive form of treatment as well [4].

## 2. Materials and Methods

Databases contained within PubMed were employed for identifying multiple empirical and review articles in the relevant literature. The keywords employed for the searches were “combinatorial polyphenols”, “cancer”, “epigenetics”, “curcumin”, “resveratrol”, and “epigallocatechin gallate”. PubMed was used for comprehensive searches for relevant research references as well as review article references. Although descriptive statistical analysis and meta-analyses were not employed for the searches.

## 3. Polyphenols

**Figure 1:**
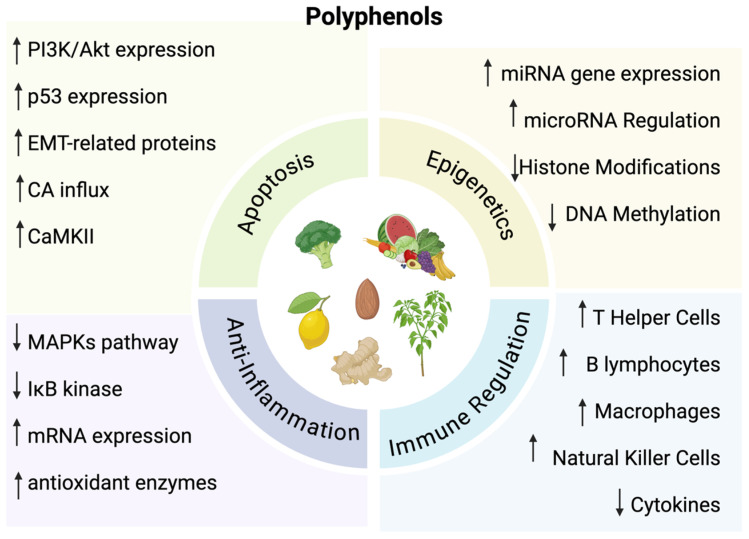
↑ = Indicates increase; ↓ = Indicates decrease. The diverse functions of polyphenols. Created in BioRender. Lab, T. (2024) https://BioRender.com/l10i067 (accessed on 7 December 2024) [7,8,9,10].

Polyphenols are natural compounds derived from plants. They play an important role for plants by assisting in the attraction of pollinators, defense for ultraviolet radiation, and protection against microbes [5,6]. These compounds are also found in various diets, particularly fruits, vegetables, and tea [3]. They enhance the quality of foods by affecting color, odor, and binding to macromolecules to reduce food digestibility [6]. While these compounds play a prominent role in plants and diets, there have been numerous studies demonstrating an ability to influence oxidative and inflammatory stress, as well as inhibit the progression of cancer through inhibition of cell cycle events and apoptosis induction [7,8,9,10]. Specifically, polyphenols affect pathways of immune regulation, epigenetic mechanisms, oxidative stress pathways, apoptosis, and inhibition of metastasis (Figure 1).

### 3.1. Epigenetic Mechanisms

Polyphenols have specific effects on epigenetic mechanisms of the human body. This can include DNA methylation, histone modifications, and posttranscriptional regulation by microRNAs [11]. These epigenetic pathways have a significant role in the development of cancer, which is why polyphenols can be synthesized to be utilized as a form of treatment for cancer [12]. For instance, certain polyphenols can inhibit DNA methyltransferases, which can lead to changes in the gene regulation of tumor suppressors and oncogenes to prevent tumor formation [13,14,15]. Polyphenols also have the ability to inhibit histone deacetylase activity while increasing the acetylation of lysines to re-activate the expression of tumor suppressor genes and other previously suppressed genes [16]. In addition, through targeting miRNAs, polyphenols can also regulate gene expression that can activate and differentiate various cell types [17].

### 3.2. Immune System Regulation

Polyphenols can also modulate the immune system. Certain polyphenols can cause an increase in immune cells, such as T helper cells, natural killer cells, macrophages and dendritic cells [18]. T lymphocytes have an important role for autoimmunity and a dysregulation or reduction in these cells causes allergies, rheumatoid arthritis, and inflammatory bowel disease [19]. Thus, having polyphenols that increase the number of T helper cells can significantly support the immune system by suppressing the prevalence of autoimmune disorders [20]. B cells are also important in the immune system and produce antibodies as well as regulate homeostasis [21]. Some polyphenols allow for increased synthesis of B lymphocytes, which then further causes an increase in antibodies such as IgG production. This antibody has a significant role in inflammatory responses which can help suppress cancer [22]. Macrophages are also involved in the inflammatory response through the production of cytokines. Polyphenols can have an inhibitory effect on cytokines through a decreased phosphorylation of extracellular signal-regulated protein kinases [23,24,25].

### 3.3. Oxidative Stress and Anti-Inflammatory Pathways

Additionally, oxidative stress is related to chronic inflammation and polyphenols can increase mRNA expression and antioxidant enzymes [26]. Cell signaling also affects inflammation through the MAPKs pathway and IκB kinase. These pathways regulate transcription factors that affect the expression of inflammatory mediators [27]. Polyphenols act by blocking the MAPKs pathway which reduces inflammation [28]. This also causes a decrease in transcription factors that are related to inflammatory processes further diminishing the effects of inflammation [27].

### 3.4. Apoptosis and Anti-Metastasis

Polyphenols can have an effect on apoptosis through a variety of mechanisms. For instance, they can diminish reactive oxygen species and phosphorylation, increase PI3K/Akt protein expression and calcium influx, and activate p53 expression and CaMKII [29,30,31,32,33]. These events can cause cancer cells to induce cell cycle arrest at the G1 phase and suppress the proliferation of cells. Polyphenols can also exhibit anti-metastatic effects by affecting epithelial–mesenchymal transition (EMT)-related proteins [34]. These proteins cause cells to change from an epithelial morphology to a mesenchymal morphology, allowing cells to migrate from their primary site to other sites of the body, leading to metastasis [35].

### 3.5. Safety of Polyphenols

A great benefit of polyphenols is that most of these compounds are derived from plants, meaning that it is considered both safe and healthy [36]. When taken at appropriate concentrations, these compounds have limited risk and side effects. Since polyphenols are natural molecules, there is a significantly reduced chance of them exhibiting genotoxic or carcinogenic side effects. This quality of polyphenols makes it even more desirable and beneficial to be utilized as a form of cancer prevention [37].

## 4. Common Polyphenols

### 4.1. Curcumin

**Figure 2:**
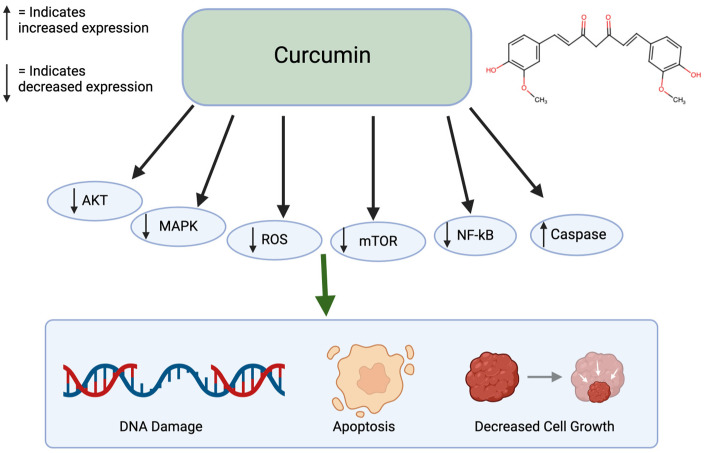
Effects of Curcumin on cancer. Curcumin affects many pathways including the AKT, MAPK, ROS, mTOR, NF-kB, and Caspase pathways. This leads to DNA damage, apoptosis, and decreased cell growth. Created in BioRender. Lab, T. (2025) https://BioRender.com/u13o398 (accessed on 5 February 2025) [39,40].

Curcumin is a polyphenol found in turmeric and ginger. It not only has potential to be used in treatment for cancer but also has therapeutic potential for multiple chronic diseases, liver disease, and arthritis [38]. Specifically for cancer, it has been shown to inhibit cancer growth, induce apoptosis, suppress cell migration, and prevent angiogenesis and metastasis (Figure 2). This polyphenol can create these effects by altering pathways, such as mTOR, PI3K/AKT, and caspase activation. When non-small lung cancer cells were treated with curcumin, it prevented cell proliferation and induced apoptosis through the upregulation of miR192-5p and suppression of PI3K/AKT signaling [39]. Treated with curcumin, ovarian cancer cells caused apoptosis and autophagy through the inhibition of AKT and mTOR signaling pathways. Similarly, curcumin-treated colon cancer cells led to a growth inhibition through the associated protein suppression [40]. With curcumin’s numerous benefits, it has low bioavailability because of poor absorption and rapid metabolism. This limits its therapeutic effect [41]. Since curcumin is lipophilic, it accumulates in fatty tissues, the liver, and kidneys, where it goes through rapid metabolic conversion. It also binds to plasma proteins. Curcumin is excreted primarily in the bile and feces and its half-life is around 1–2 h in the plasma [42]. Curcumin has been shown to have limited sub-chronic toxicity damage and has no potential mutagenic or teratogenic effects [43].

### 4.2. Resveratrol

**Figure 3:**
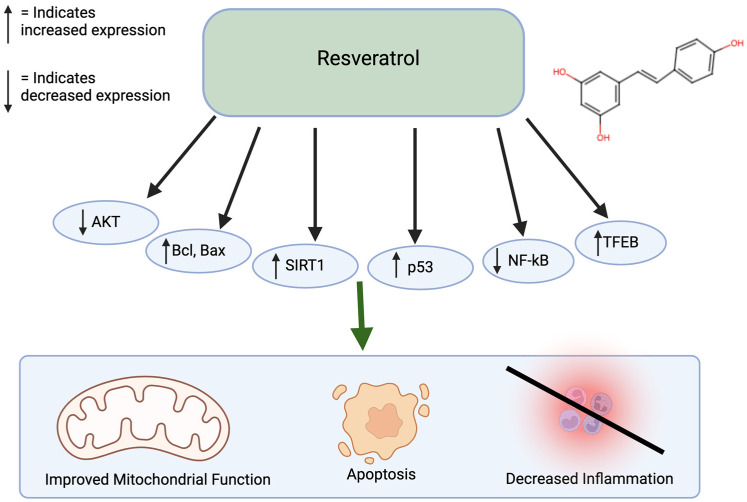
Effects of resveratrol on cancer. Resveratrol affects many pathways, including the AKT, Bcl, Bax, SIRT1, p53, NF-kB, and TFEB pathways. This leads to improved mitochondrial function, apoptosis, and decreased inflammation. Created in BioRender. Lab, T. (2024) https://BioRender.com/s56n309 (accessed on 7 December 2024) [40,49,50,51,52,53].

Resveratrol is commonly found in berries, grapes, and peanuts and has antioxidative and anti-inflammatory properties. Similar to curcumin, resveratrol also inhibits a variety of diseases, including diabetes, Parkinson’s, Alzheimer’s, as well as cancer [44]. Resveratrol treatment of colorectal cancer cells led to apoptosis and inhibition of metastasis, showing promise as an effective anti-cancer agent [45]. When used to treat gastric adenocarcinoma cells, resveratrol caused DMA damage and an increase in reactive oxygen species. Furthermore, it stimulates G2/M cell cycle arrest and increases Bcl and Bax, leading to apoptosis. When treating melanoma cells with resveratrol, there is an increase in ROS, endoplasmic reticulum stress, and increased cleaved caspase-9 levels [46,47,48]. Resveratrol combined with hepatocellular cancer cells causes growth inhibition through the regulation of p53 and PI1K/AKT pathways [40,49,50]. Resveratrol can also affect DNA methylation by decreasing the activity of DNA methyltransferases (DNMT) enzymes and change the DNA methylation patterns by decreasing levels of 5-methylcytosine. Resveratrol also influences inflammatory responses by increasing the SIRT1 and NF-kB pathways (Figure 3) [51,52,53]. Resveratrol has a low oral bioavailability. In addition, around 75% of orally ingested resveratrol is rapidly absorbed in the gastrointestinal tract [54]. Resveratrol is lipophilic and is distributed in the liver, kidney, and intestinal tissues. It goes through first-pass metabolism in the liver and intestinal cells and is metabolized by phase 2 enzymes. Finally, it is excreted in the bile and feces. Resveratrol is relatively safe, as it has minimal adverse effects at low-to-moderate doses and is well-tolerated at high doses. Resveratrol also has low toxic effects in animal studies [55].

### 4.3. Epigallocatechin Gallate

**Figure 4:**
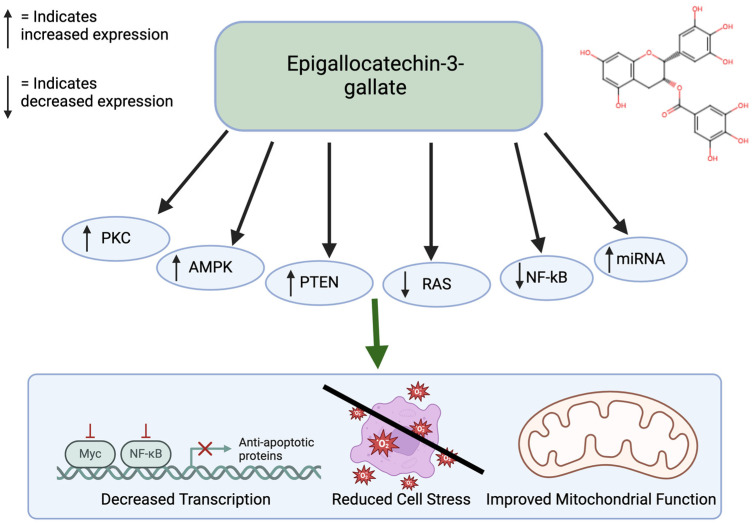
Effects of EGCG on cancer. EGCG affects many pathways, including the PKC, AMPK, PTEN, RAS, NF-kB, and cAMP pathways. This leads to improved mitochondrial function, reduced cell stress, and decreased transcription. Created in BioRender. Lab, T. (2024) https://BioRender.com/s56n309 (accessed on 7 December 2024) [57,58].

Epigallocatechin gallate (EGCG) is a prominent antioxidant in green tea. This polyphenol shows significant antiproliferative effects and apoptotic effects. Specifically, ECGG has been shown to inhibit colon cancer [56]. Through experimental methods, it has been found that ECGG inhibits cellular growth of colon cancer cells by stimulating early apoptosis and cell cycle arrest, particularly in cancer cells with functional p53 [57]. ECGG also has anti-tumor effects in the ability through activation of the AMPK pathway to inhibit cell-growth-promoting anabolic pathways [58]. ECGG has also been shown to act as an inhibitor of DNMTs to reactivate silenced tumor suppressors, thereby affecting cancer-related DNA hypermethylation. It also promotes tumor suppression by increasing the PTEN gene. EGCG influences cell death by regulating the PKC and RAS pathways (Figure 4). In addition, it exerts anti-cancer effects through miRNA silencing. miRNAs that do not function properly lead to decreased programmed cell death, upregulation of telomerase, and cellular proliferation, which can facilitate tumor formation [57,59]. EGCG has low bioavailability. EGCG is hydrophilic and is present in lipid products. There are structural modifications through esterification that allow it to be absorbed better [60]. EGCG is also considered safe in moderate doses but at higher doses it could cause adverse effects. High doses can cause liver toxicity [61].

### 4.4. Other Polyphenols

Other common polyphenols include quercetin, which increases apoptosis in cancer cells by activating caspase-3 and inhibiting the phosphorylation of signaling pathways, such as mTOR, Akt, and ERK. Quercetin also has the ability to prevent angiogenesis by targeting the VEGRF-2-mediated angiogenesis pathway and thus reducing tumor growth. Quercetin’s ability to inhibit EMT and STAT3 signaling were reported to prevent metastasis [62]. Kaempferol also has been shown to have anti-cancerous properties. Specifically, studies show that it inhibits the growth of breast cancer cells. Kaempferol can arrest the cell cycle at the G2/M phase and induces apoptosis by suppressing the Bcl2 protein and increasing the Bax protein. In addition, it inhibits the migration of breast cancer cells by regulating EMT [63].

Polyphenols when used in combination can have synergistic effects that can be used as better treatment options than utilizing the polyphenols individually. By using the appropriate dosages of each polyphenol, toxicity can be avoided. If the dosages are too high, potential side effects could occur, including interference with iron absorption, genotoxic effects, and pro-oxidant activity [6].

## 5. Combination of Polyphenols

### 5.1. Curcumin in Combination with Other Molecules

#### 5.1.1. Curcumin and EGCG

Curcumin and ECGG have been studied together to explore their synergistic effects. For example, one study showed the combinatorial inhibitor effects of both compounds in colorectal carcinoma [64]. Specifically, it blocked the JAK-STAT3 signaling pathway which is overactivated in colorectal cancer cells. This blocks the transition of normal endothelial cells (NECs) to tumor endothelial cells. When NECs were treated with both compounds in combination there was a significantly greater inhibitory effect than when the compounds were used individually. mRNA and protein levels of proteins such as JAK, STAT3, TEC markers, and IL-8 were reduced in the treatment group versus the control group. Collectively, this contributes to a reduction in cell migration, invasion, and viability [64]. While this study demonstrates promising results in the combination treatment for the inhibition of colorectal cancer cells, it primarily focuses on the JAK/STAT3 signaling pathways. There are other angiogenic pathways or factors that also contribute to the NEC to TEC transition that can lead to colorectal cancer. The combination treatment can be explored to determine if it induces a suppressive effect on these pathways as well.

The synergistic effect of curcumin and EGCG has also been investigated in prostate cancer. Combination treatment resulted in a greater shrinkage of cell morphology and karyorrhexis compared to treatment with individual molecules. In addition, the expression of p21 protein was increased, which leads to cell cycle arrest and inhibition of transcription and the inhibition of *p*-Rb, which leads to increased cell death. Curcumin and ECGG combination affect multiple cell cycle target pathways, which causes an arrest of the cell cycle in different stages, such as the G2/M phase and the S phase [65].

#### 5.1.2. Curcumin and Resveratrol

There have been studies showing the combination of resveratrol and curcumin in colorectal cancer cells. Combination treatment with both compounds has a greater inhibitory effect of the colorectal cancer cells lines Caco-2 and DLD-1 than when treated with the compounds individually. In addition, when treated with both compounds, there were differences in gene expression for *PMAIP1*, BID, *ZMAT3*, *CASP3*, and *FAS*, where the gene expression is decreased, causing an increase in apoptosis [66]. The combination treatment resulted in a 300% higher apoptosis in comparison to treatment with the compounds alone. In addition, both compounds act on cancer cells independent of p53 status, meaning that they exert effects on p53 positive and negative cells. Treatment also led to cell cycle arrest at the G2-M phase, where there is a decrease in the number of cells in the G2-M phase and an accumulation of cells in the S phase. The combination treatment leads to an activation of EGFRs and IGF-1R in colon cancer cells.

Studies have been performed in SCID mice with colon cancer, where the combination of resveratrol and curcumin caused a 50% inhibition in tumor growth with limited systemic toxicity or adverse effects. Daily gavaging of either compound individually led to a 40–45% inhibition in growth, while the combination causes a 70% inhibition [67]. This study presents significant results in vivo but does not explore the effect on in vitro cell lines, so it does not show how the combination affects human-derived cell lines.

An additional study demonstrated how the combination of curcumin, resveratrol, and benzopyrene produces an increased chemopreventative response by maintaining proper zinc levels [68]. Zinc plays an important role in prostate development and suppresses the angiogenic and metastatic potential of malignant prostate cells. It also increases p21 levels and modulates Cox-2 [68,69].

#### 5.1.3. Curcumin and Tocotrienol-Rich Fraction Vitamin E

There have been reports indicating a significant combinatorial effect of essential turmeric oil-curcumin (ETO-Cur) and tocotrienol-rich fraction vitamin E (TRF) isomers in colon cancers [70]. The combination of ETO-Cur and TRF showed greater inhibition than when the compounds were administered individually on both the colon cancer cell lines HCT-116 and HT-29 and HCT-116 xenograft mouse model, demonstrating that this effect is present in vitro and in vivo. In addition, the combinatorial treatment was seen to have an effect in the gut microbiome, which affects the growth and progression of colon tumors. Using 16S rRNA sequencing, it was shown that the number of microbial bacterial species increased in the SCID mice in comparison to the control. Not only did the number increase, but the richness and diversity increased 44% in SCID mice versus the control. Specifically, it was shown that probiotic bacteria Bifidobacteria and Lactobacillus increased as well as beneficial bacteria, such as Clostridium IV, which has anti-inflammatory effects, in comparison to the control [70]. This study presents results both in vitro and in vivo, which shows various effects of the combination in a clinical and basic science aspect. Utilizing a mice model presents a challenge where the gut microbiome is highly variable, as it is influenced by diet, environment, and genetics. Thus, the microbial changes that were observed in mice may not replicate the diversity and complexity of the human gut microbiome. In addition, while the changes in microbial composition were observed, functional analysis of the gut microbiota was not conducted, which limits the understanding of how microbial metabolites can contribute to tumor inhibition.

#### 5.1.4. Curcumin and 5-Flurouracil (5-FU) and Oxaliplatin

Combinations of curcumin with chemotherapy agents have also been reported to enhance cancer treatment efficacy. Curcumin with 5-FU and oxaliplatin (FOLFOX) was given to patients with colorectal liver metastases [71]. The trial showed that the combination decreased the growth of primary cancer stem cells (CSC) spheroid numbers by 80% in comparison to these agents used alone. ALDH1Ai staining indicated that there were also decreased pluripotent stem cell markers, such as Oct3-4, AFP, and HNF/FOxAe, disrupting important stem cell pathways that contribute to the growth of cancerous cells. The combination overall resulted in increased proapoptotic effects and a decreased proliferative index. Although there were some side effects, the treatment was generally safe, as 41.7% of patients reported no adverse events [71]. While a significant amount of patients did not experience serious side effects, this could be due to variations in individual metabolism, tumor biology, or prior treatment history that can lead to conflicting findings. However, a great strength of this article is that it incorporates a mix of in vitro experiments and patient clinical trials, showing a multifaceted evaluation of curcumin’s effects.

#### 5.1.5. Curcumin and Doxorubicin

Doxorubicin is another chemotherapeutic agent that is used to treat many cancers, such as breast cancers, by intercalating with DNA, releasing reacting oxygen species, and negatively affecting DNA damage repair. The combination of the chemotherapeutic agent with curcumin resulted in reducing the survival of breast cancer cells. Flow cytometry showed an increased cytotoxicity for breast cancer cells in comparison when the combination was used [72].

In addition, curcumin can further amplify the immune and cardioprotective properties of doxorubicin [73]. This combination has also been shown to affect leukemia and lymphoma cells. Specifically, it inhibits mRNA expression of MDR1 and Bcl-2 in CML-inhibiting cancer growth and activating apoptosis. Furthermore, the synergistic effect of curcumin inhibits drug resistance of Doxorubicin so that it retains in the nucleus longer [74].

Curcumin can also improve the stability and deliver doxorubicin slowly into the system for the treatment of lung cancer. In vitro studies through MTT assays showed that the combination induced apoptosis by 20.02% in comparison to 9.57% and 11.33% for curcumin and doxorubicin administered individually, respectively. The combination also inhibited angiogenesis, as zebra fish embryos treated with both compounds had defective vascular formation and intersegmental vessels failing to form. In vivo studies of mouse models showed that tumor was decreased more for combination treatment than individual treatment as well as an increase in positive nuclei indicating apoptosis [75]. This study observes the inhibition of doxorubicin and curcumin on the angiogenesis pathways in zebrafish and mouse models. However, this may not apply to other species due to the species-specific variations in angiogenesis pathways among tumors. In addition, this study highlights a micelle-based delivery system to enhance water solubility; however, this may be difficult to achieve in therapeutic plasma concentrations due to curcumin’s poor bioavailability. Nevertheless, the study presents detailed analysis of micelle size and morphology to support the reliability of the delivery system. In addition, it demonstrates the synergistic effects through various aspects, such as in apoptosis, tumor growth inhibition, and antiangiogenics, to support how it can be utilized as an anti-cancer treatment.

#### 5.1.6. Curcumin and Paclitaxel

Paclitaxel is another chemotherapeutic agent that is used as a mitotic inhibitor [76]. Synergistic combinations of curcumin and paclitaxel induced a downregulation of NF-kB activation, which led to an inhibition of the nuclear translocation of the p65 subunit. Cells treated with both compounds also had an over-expression of WT Akt. The two events of the inactivation of NF-kB and activation of Akt were independent events. Through Annexin V/PI staining, it was shown that the combination also induced apoptosis and G2/M cell cycle arrest in cervical cancer cells [77]. There was also an upregulation of the survivin protein that is activated by Akt. Immunoblotting demonstrated that the MAPK pathway had a role in the synergism between curcumin and paclitaxel. Finally, Western blots showed an upregulation of the Bcl-2 protein that is responsible for apoptosis. In addition, the usage of curcumin reduces the harmful side effects of paclitaxel, making this combination a more available chemotherapeutic treatment [77]. This study demonstrates how the combination affects the apoptotic pathway for Bcl-2 and Bax but does not explore the broader apoptotic pathways and alternative cell death mechanisms that could also be affected by the combination. In addition, there are no therapeutic explorations of the combination with p53 and p21 through utilizing clinical models.

A summary of the combinatorial effects of curcumin with other polyphenols is provided in Table 1 and Table 2 below.

### 5.2. Resveratrol in Combination with Other Molecules

#### 5.2.1. Resveratrol and Piperine

Piperine has been shown to increase the bioavailability of resveratrol. Piperine is an alkaloid found in black pepper. The degree of exposure for resveratrol was increased to 229% when piperine was also used in combination [78]. This is very beneficial because resveratrol cannot exert a significant effect for anti-cancer mechanisms because of its rapid metabolism. By utilizing piperine alongside resveratrol, it inhibits its metabolism allowing resveratrol to have a longer impact [78].

#### 5.2.2. Resveratrol and Quercetin

Resveratrol and quercetin demonstrated synergistic effects for skin cancer. A cell cytotoxicity study indicated that the combination treatment had a lower IC50 value, indicating an increased cytotoxic effect towards skin cancer cells [79]. This also demonstrated that the combination treatment was helpful in overcoming oxidative stress and inflammation. A wound-healing assay showed that quercetin-resveratrol treatment inhibited the migration of cells as the cells in the control group moved to the scratch edges and healed the wound, whereas the experimental group did not have any migration of cells or healing of wounds. This supports the fact that the combination treatment contributes to anti-metastatic effects [79].

Resveratrol and quercetin have also been shown to affect glioma cancer [80]. MTT assays showed that when used in combination, the cells decrease by 80%, which is significantly less than when the cells are treated with the compounds individually. When treated with resveratrol, there is a reduction in the number of cells by 25%, and when treated with quercetin there is a reduction in the number of cells by 54%. The synergistic effect of these compounds also caused a strong activation of caspase 3/7 activity, which leads to increased apoptosis. These compounds decreased the phosphorylation of AKT, which reduces cell growth, proliferation, and survival [80]. Although the study showed good results, it used relatively high concentrations of both resveratrol and quercetin, which may not be achievable in clinical settings. It also may be toxic at these higher doses. In addition, there is also limited cell viability testing, as the studies rely heavily on MTT assays, which may not show all aspects of cytotoxicity. Other methods, such as flow cytometry, may also be utilized.

There has also been a study on resveratrol, quercetin, and ellagic acid on leukemia cells. MTT assays indicated an increased additive effect when all three compounds were used, as this led to decreased cell viability and cell number. Isolbolographic analysis showed increased caspase-3 activity when all three compounds were used when compared to using the compounds individually [81].

Resveratrol and quercetin have shown to also inhibit neointimal hyperplasia by decreasing serum amyloid A and soluble vascular cell adhesion molecules. Thus, it reduces restenosis, which also decreases the risk of developing cancer [82].

#### 5.2.3. Resveratrol and Pterostilbene

Resveratrol and pterostilbene creates a synergistic effect on ERα-negative breast cancer [83]. Western blots show that the combinatorial treatment creates an increase in ERα protein expression compared to using the compounds individually. In addition, these compounds together cause a decrease in acetyl-H3 and acetyl-H4 histone markers in the promoter region, which represses transcription. There is also a significant increase in HAT activity and a decrease in HDAC activity for combination treatment, whereas there is no change in activity for individual treatments. Moreover, there is no change in the control cell line, indicating that this treatment only targets the cancer cell lines. Similar to HDAC activity, there is also a decrease in DNMT activity in breast cancer cell lines. Overall, this combination treatment supports the appropriate epigenetic modifications to decrease the proliferation of cancer cells [83]. The study demonstrates an optimal does of 15 μM of resveratrol and 5 μM of pterostilbene; however, there is limited exploration of the dose–response relationship. Further experiments could determine the minimal dose that has an effect on cancer cells and the maximal dose to be used where there is no toxicity. The study primarily focuses on HDAC and DNMT activity but not other epigenetic factors.

Another study demonstrated how this combination induces apoptosis in breast cancer cells with no apoptotic effects on the control cells, as well as cell cycle arrest in the G2/M phase and S phase [84]. Phospho-H2AX is a marker for DNA damage and repair mechanisms. Cancer develops through an increased expression in Phospho-H2AX, which leads to increased DNA damages and mutations [85]. Western blotting showed that combinatorial treatment leads to a decrease in Phospho-H2AX levels, which prevents the formation of cancer cells. The human telomerase reverse transcriptase (hTERT) is also downregulated during combinatorial treatment, as depicted through RT-PCR and Western blot analysis [84].

#### 5.2.4. Resveratrol and Arsenic Trioxide

Resveratrol and arsenic trioxide together have been studied in human lung adenocarcinoma and hepatocellular carcinoma cells. MTT assays demonstrated that the combination treatment led to decreased cell viability compared to individual treatments, causing an inhibition of colony formation and cell proliferation. Combination treatment increases Nrf2 activation, which leads to an ROS accumulation. This ROS accumulation then leads to increased apoptosis [86]. Apoptosis is also increased in cells treated with the combination, as depicted by Western blots that show the elevated caspase 3 activation. ROS induces ER stress as well as several ER-stress-related proteins, such as GRP 78, CHOP, and caspase 12, which were detected after treatment with both compounds. Mitochondrial dysfunction was also demonstrated, which further contributes to the apoptosis of cancer cells [86]. This study demonstrates how resveratrol enhances Nrf2 activation, which can lead to ROS and cytotoxicity. However, Nrf2 has also been shown to act as a protective mechanism, which can lead to contradictions about its role in cancer therapy. In addition, the study illustrates how synergism can cause ER stress and mitochondria dysfunction, but there can be differences in these pathways across tumors. This presents variable outcomes depending on the tumor. The study presents validation of combinatorial treatment into cell lines for lung and liver cancer. While most studies focus on one specific cell subtype, this study effectively shows the translational potential of the compounds in two different cancers.

#### 5.2.5. Resveratrol and Melatonin

Melatonin is not only involved in the regulation of the circadian rhythm but is also an antioxidant and anticarcinogenic agent for cancer, specifically targeting breast cancer. The effects of melatonin and resveratrol in a rat model with mammary carcinogenesis have been investigated [87]. The results indicated a decreased tumor incidence of 17% for the rats when given the resveratrol–melatonin combination diet. There was a decrease in the number of invasive carcinomas when rats were given the combination, whereas when treated with just resveratrol alone, there was no effect on tumor incidence and when melatonin was administered alone, there was an increase in the number of invasive tumors [87].

#### 5.2.6. Resveratrol and Grape Seed Proanthocyanidins

Another study examined the effects of resveratrol with grape seed proanthocyanidins (GSPs) in breast cancer [88]. MTT assays indicated that the combination treatment inhibited cancer cell lines MDA-MB-231 and MCF-7 more than when the cell lines were treated with the compounds individually. For instance, after 48 h of treatment with GSPs individually, there was a 9–19% decrease in cell viability and after 48 h of treatment with resveratrol individually, there was a 15–42% decrease in cell viability. However, when treated in combination, there was a 44–79% decrease in cell viability after 48 h of treatment. Clonogenic assays were performed to analyze the long-term anti-carcinogenic effects on cell proliferation on the cancer cell lines. The results show that there were significant reductions in colony formation when the cells were treated with both compounds instead of each compound individually. The effects on apoptosis from the combination treatment was analyzed through flow cytometry and observing cell density. There was a decrease in cell density from the combination treatment in comparison to individual treatment from each compound. From flow cytometry, results indicated that the combination treatment induced apoptosis by 21.8% compared to 3.4% and 4.1% from grape seed polyphenols (GSPs) and resveratrol, respectively. Apoptosis can also be measured by observing the regulation of the pro-apoptotic protein Bax and the anti-apoptotic protein Bcl-2 expression. Specifically, an upregulation of Bax and downregulation of Bcl-2 signifies increased apoptosis. Western blot analysis showed that combination treatment almost quadrupled the expression of Bax and decreased Bcl-2 expression by 70%. Finally, HDAC and DNMT activity assays indicated that combination treatment decreased the activity of both epigenetic modulators compared to treatment with the compounds individually [88]. The therapeutic efficacy of the combination could be explored further. One way to do this would be to have a biomarker analysis to evaluate its therapeutic potential at the molecular level. This would help determine if the combination could be used as a treatment in clinical settings. It would also show if cells develop any resistance to the combination.

#### 5.2.7. Resveratrol, EGCG, and γ-Tocotrienol

EGCG, another common polyphenol used to treat cancer, was also investigated alongside resveratrol as well as γ-tocotrienol [89]. γ-tocotrienol is a vitamin E analog, which has shown to have anti-proliferative effects on breast cancer cells [90]. Cell number, cell viability, colony formation, and morphological changes were analyzed using different concentrations and combinations of the compounds individually, in pairs, or all three combined together. Cell number was most decreased when 10 μM of resveratrol and 10 μM γ-tocotrienol was used, but 10 μM of all three compounds still demonstrated a significant decrease in cell number. Cell viability was most reduced with treatment of 10 μM of γ-tocotrienol alone, while the treatment of all three compounds had little effect on cell viability. Colony formation on the other hand was most inhibited when 10 μM of all three compounds was used. The addition of all three compounds for the above study created the most morphological change. Cell cycle arrest was also analyzed through conducting Western blot analysis of the retinoblastoma Rb/E2F protein complex, as it plays an important role in the G1 checkpoint. There was significant inhibition of the Rb/E2F expression with the resveratrol and γ-tocotrienol combined treatment. There was also inhibition of Rb but not E2F with combined treatment consisting of EGCG with either resveratrol or γ-tocotrienol. However, when all three compounds were administered, the inhibition was less pronounced. Cyclin D1 and cdk4 play a role in regulating the Rb/E2F complex, so Western blot analysis was also conducted to detect expression of these proteins as well [89].

The combined treatment of resveratrol and γ-tocotrienol showed the most prominent effect on cyclin D1 and cdk4 expression in comparison to the combination of other compounds. The ratio of Bcl-2 and Bax was also analyzed as an indication of apoptosis. The most significant increase in the bax/bcl-2 ratio was seen in treatment with resveratrol and γ-tocotrienol with the ratio being 2.30. However, the second highest increase in ratio was seen in combined treatment, with all three compounds yielding a ratio of 1.35. Finally, changes in activities of the antioxidant enzymes SOD, catalase, and NQO1 were analyzed. The combination of all three compounds did not lead to much of a change in activity for the SOD and catalase enzymes but it led to a great increase in activity for the antioxidant enzyme NQO1. All these results demonstrate that there was a prominent synergism between resveratrol and γ-tocotrienol; however, the combination of all three compounds still demonstrated a good amount of synergism of anti-cancer activity [89].

#### 5.2.8. Resveratrol and Oxaliplatin

Oxaliplatin is a platinum compound that is used as part of chemotherapy for advanced colorectal cancer, as it blocks the replication and transcription of DNA [91]. The efficacy of oxaliplatin combined with natural compounds, specifically resveratrol, was investigated [92]. In vitro cytotoxicity analysis demonstrated that there was a greater decrease in cell viability when cells were treated with resveratrol and oxaliplatin together than alone. In vivo therapeutic efficacy analysis through observing tumor volume was also evaluated. The results show that the combination exhibited the strongest tumor growth inhibition, as the tumor weight decreased significantly compared to treatment with compounds individually. This indicates that the combination caused a suppression of tumor growth. While the in vivo model demonstrated a suppressive effect with the combination treatment, the sample size for the model was small. This limits the statistical power and generatability of the results.

The expression of α-SMA and CUGBP1 in tumor tissues was also studied through IHC [92]. α-SMA is a protein part of cancer-associated fibroblasts, and CCGBP1 promotes cell proliferation [93]. IHC results show that there was a decrease in expression of α-SMA, which indicates an improvement in tumor fibrosis, as well as a decrease in CCGBP1 protein, showing a weakening of liver fibrosis. Tumors also utilized myeloid-derived suppressor cells (MDSCs) to support their immune-escaping abilities. The combination treatment led to a reduction in MDSCs, which demonstrates a stronger impact on tumor microenvironment, a reduction in fibrosis, and inhibition of the development of tumors [92].

#### 5.2.9. Resveratrol and 5-Flurouracil (5-FU)

5-FU is a common treatment for patients with liver cancer, and resveratrol has been shown to prevent tumor growth and metastasis for many cancers, including liver cancer. This study investigated the combination of resveratrol and 5-FU in liver cancer. The results indicate that resveratrol combined with 5-FU had synergistic suppressive effects on transplanted liver cancer in mice. For instance, when 10 mg/kg RES was treated in combination with 5mg/kg of 5-FU, the inhibition was 50%. However, when mice were administered 5 mg/kg of 5-FU alone, the inhibition rate was 28.4%. Resveratrol was also shown to induce the S phase arrest of the liver cancer cell lines, enhance the anti-tumor effect of 5-FU, and antagonize its toxicity [94]. The combination of 5-FU and resveratrol was also observed in skin cancer [95]. The results show that the combination treatment increased apoptosis, as resveratrol enhanced 5-FU’s ability to intervene in DNA synthesis through blocking thymidylate activity synthase and promoting the expression of p53 genes to increase apoptosis. Multiple experiments in texture analysis, in vivo safety studies, and macro-tumor analysis were repeated multiple times. This enhances the reliability of the results from the study. In addition, results had *p* values of less than 0.001 that show significant results in tumor volume reduction and inflammatory cytokines support the strength of the conclusions.

The combination also led to improved antioxidant activity, as it accelerated DNA regeneration and removed DNA mutation. Resveratrol used in combination with 5-FU resulted in synergistic anti-inflammatory effects as well as down regulation of NF-kB activity and inhibition of phosphorylation of STAT-3, which is a pro-inflammatory transcription factor that when phosphorylated promotes tumor proliferation and survival [95].

Another study also investigated the effects of both resveratrol and 5-FU in skin cancer. This study suggested that there was a greater delivery of drugs, as it demonstrated a prolonged release of drugs and permeation in tight junctions of the skin, allowing the drug to cross the skin barrier and to be an effective chemotherapeutic treatment for skin cancer [96].

#### 5.2.10. Resveratrol and Endoplasmic Reticulum (ER) Stress Activators

ER stress activators tunicamycin and thapsigargin were used in combination with resveratrol to observe its anti-tumor effects on gastric tumors. ER stress leads to apoptosis [97]. Tunicamycin and thapsigargin specifically work by inhibiting the synthesis of glycoproteins and regulating calcium homeostasis. The combination of both compounds led to reduced cell viability and increased levels of other proteins related to ER stress, such as Bip and CHOP, proteins related to apoptosis such as Bak, LC3-II, which is protein for autophagy, and p62, which is a protein for necrosis [98,99]. There were also decreased levels of the migration proteins MMp2 and MMp9 [97]. The study focuses primarily on ER stress and its anti-cancer effects. However, ER stress can also have protective and pro apoptotic effects. The study does not fully exposure whether the induction of ER stress could also lead to unintended effects, such as drug resistance. The doses used in the study are also relatively high, which may be toxic in human clinical settings.

#### 5.2.11. Resveratrol and Tamoxifen

4-(E)-{(4-hydroxyphenylimino)-methylbenzene,1,2-diol} (HPIMBD) and 4-(E)-{(*p*-tolylimino)-methylbenzene-1,2-diol} (TIMBD) are novel analogs of resveratrol that can inhibit breast cancer cells. These analogs were combined with tamoxifen to observe its effect on the proliferation of breast cancer cells [100]. The combination treatment did not have an effect on the proliferation of control breast epithelial cancer cell lines MCF10A and MCF-10F. However, the combination treatment had an effect on breast cancer cell lines MCF-7, T47D, MDA-MB-231 and MDA-MB-468, as shown through MTT assays. Tamoxifen alone did not have any significant inhibition of breast cancer cells, while the resveratrol analogs alone had 50% inhibition of cells. The combination treatment created a 70–80% inhibition on proliferation, which demonstrates a synergistic effect. Apoptosis and necrosis assays showed that the combination had a significant increase in late apoptosis than the compounds individually. Beclin-1 is an autophage inducer that is expressed when breast cancer cells are treated with the resveratrol analogs alone [101]. However, when the cells were treated with the combination, it resulted in a larger expression on beclin-1 of about 2-fold as well as an increased expression of LC3BII by about 5-fold [100]. The combination also led to an inhibition of expression of levels of ERα and c-Myc. For ERα, tamoxifen did not inhibit the expression much, and for the resveratrol analogs used alone, it inhibited the expression by 50–60%. When used in combination, the synergistic impact inhibited the expression by around 70–90% [100]. Results in this study demonstrate that there was no toxicity in non-neoplastic breast epithelial cells despite using high doses. However, resveratrol has been shown to exhibit toxicity in normal epithelial cells, which can present a potential conflicting result. However, this study tests the combination across multiple breast cancer lines, such as ER-positive and ER-negative cells enhancing the validity of the results. In addition, the study shows how the combination induces apoptosis, autophagy, and ERα suppression, demonstrating how it negatively affects cancer growth in multiple ways.

#### 5.2.12. Resveratrol and Cytochalasin D

Cytochalasin D (CytD) and focal adhesion kinase inhibitor (Fak-I) are pharmacological inhibitors that affect the cell viability and migration of colorectal cancer cells [102,103]. Cell viability decreased more when the cells were treated with the combination of both compounds rather than each compound individually [104]. Resveratrol alone induces the histone deacetylase Sirt1, which suppresses colorectal cancer proliferation and metastasis. However, when resveratrol was combined with the FAK-I or CytD, it led to a decreased expression of Sirt1 protein. On the other hand, FAK is involved in cancer cell survival and invasion, and the combination treatment significantly reduced FAK and focal adhesion clusters. The combined treatment also led to increased apoptosis, as IHC demonstrated an increase in caspase-3 expression. The evaluation of colony formation showed the effects of these compounds on the tumor microenvironment. These results show that the combined treatment resulted in the greatest invasion-inhibition ability compared to treatment with the compounds singly administered. FAK functions through integrin signaling functions and resveratrol is known to decrease B1 integrin levels, thereby inhibiting the phosphorylation of FAK [105]. Western blotting showed that the combination treatment suppressed integrin expression, which reduces the phosphorylation and activation of FAK. Finally, the combination treatment led to a greater suppression of NF-kB, which is also involved in FAK activity [104].

#### 5.2.13. Resveratrol and Salinomycin

Salinomycin is a Wnt inhibitor and HLY78 is a Wnt activator, both of which affect breast cancer cells [106]. When observing the combinatorial effects of resveratrol and Wnt activator HLY78, it was shown that resveratrol at low dose did not significantly affect cell viability, HLY78 had some toxicity, and the combination had minimal effects [107]. However, the combination of salinomycin and resveratrol had significant effects on cell viability, exhibiting an additive effect on breast cancer cell lines. Western blotting analysis of the Wnt signaling pathway revealed that the combination treatment downregulated proteins involved with the Wnt signaling more than when the cells were treated with compounds individually. Western blotting also showed that the combination treatment downregulated CDK2 and CDK4 proteins, which leads to G phase cell cycle arrest. However, the combination resulted in a decrease in the expression of pro-apoptotic proteins PARP, caspase-8, and caspase-9 [107]. While the combination consistently downregulated the Wnt signaling in all cell lines, the salinomycin effect was less pronounced in some cell lines. In addition, the study focuses on only a few breast cancer subtypes, so it would be beneficial to determine if the combination also exhibits these effects on other subtypes.

#### 5.2.14. Resveratrol and Pemetrexed

Pemetrexed demonstrates anti-cancer activity for non-small cell lung cancer [108]. When pemetrexed and resveratrol were administered together in ratios of 1:5 or 1:2, it resulted in the loss of cell viability demonstrated. The combination also decreased ERCC1 mRNA expression and stability, which enhances resveratrol’s cytotoxic effects [109]. Through utilizing a host cell reactivation assay, luciferase activity was measured, which correlated to DNA repair activity. The combination enhanced luciferase activity, which indicates an improved DNA repair ability. However, the suppression of ERCC1 downregulates DNA repair abilities [109]. The study clearly demonstrates how the combination has downstream effects for the MAPK-ERCC1 pathway, but it does not look at other pathways, such as apoptosis or autophagy. It also does not explore the long-term effects of the combination treatment.

An evaluation of pemetrexed and resveratrol on lung cancer were conducted [110]. Through MTT assays, it was demonstrated that the combination led to increased cytotoxic effects on lung cancer cells while maintaining minimal cytotoxicity to the control cell line. The IC50 value for the combined treatment was 4.5 ug/mL, which was lower than the IC50 values for PMX and resveratrol individually, which were 7.9 and 8.4 ug/mL, respectively. The combination also demonstrated sustained release, as there was greater cytotoxicity after 48 h compared to 24 h. In vivo studies using mice was also conducted to evaluate the effects of the combination treatment. After 25 days, the greatest lung tumor weight was for the combination treatment compared to individual treatment. In addition, there were also fewer malignant lesions for the combination treatment [110].

#### 5.2.15. Resveratrol and Clofarabine (CIF) and All-Trans Retinoic Acid (ATRA)

CIF has anti-cancer activities, as it is able to change DNA methylation marks [111]. ATRA is a nutrient that can regulate epigenetic machinery similar to resveratrol [112]. The effects of all three compounds for chronic myeloid leukemia (CML) were conducted in such a way that specifically evaluated changes of expression for DNMT1 and CDKN1A as well as the expression of tumor suppressor genes *PTEN* and *RARB* [113]. CIF and resveratrol had the most significant increase in apoptosis as well as caspase-3 activation. However, ATRA alone or in combination did not produce as much apoptosis or caspase 3- activation. The most significant decrease in DNMT1 expression was the combination of CIF and ATRA, which led to a decrease of 50% in DNMT1. This combination also enhances the CDKN1A protein that regulates DNA damage response and decreases DNA methylation of tumor suppressor genes. The CIF and resveratrol combination led to a 50% hypomethylation of the PTEN tumor suppressor promotor. In addition, this combination upregulated the PTEN expression. The same pattern was observed for CIF and ATRA combination. RARB is another tumor suppressor that negatively regulates the PI3K/AKT signaling pathway [114]. CIF and resveratrol led to a 60–70% increase in RARB expression and a demethylation of the promotor. This was also seen in the CIF and ATRA combination as well [113]. This study demonstrates a DNMT1 downregulation in CML cells; however, there have been instances in which DNMT1 is upregulated in leukemia [115]. This study does highlight the potential translational impact of CIF and ATEA in targeted epigenetic dysregulation in CML, which strengthens its clinical importance.

A summary of the combinatorial effects of resveratrol with other polyphenols is provided in Table 3 and Table 4 below.

### 5.3. Epigallocatechin-3-Gallate (EGCG) in Combination with Other Molecules

#### 5.3.1. EGCG and Sulforaphane

Epigallocatechin-3-gallate (EGCG) is found in green tea polyphenols and has been shown to have anti-cancer effects through initiation of cell cycle arrest as well as preventing tumor metastasis. It also inhibits cancer through epigenetic mechanisms such as preventing hypermethylation, reactivating tumor suppressors, and facilitating histone modifications [116]. Sulforaphane (SFN) is found in broccoli sprouts and can also reduce the risk of developing many cancers through similar mechanisms as EGCG [117]. There have been studies examining the combinatorial effects of both EGCG and SFN.

For instance, one study evaluated the combinatorial effects of both compounds in breast cancer [118]. When treating MDA-MB-231 and MDA-MB-157 cells with 20 uM EGCG and 10 uM SFN, there was a synergistic effect, as they inhibited cell proliferation while also preventing toxic effects on normal cells. The combination also led to an increased ERα re-expression in ER-negative breast cancer cells when compared to the effects of the individual compounds. Moreover, the effects of this combination were very similar to the epigenetic effects exhibited by demethylation agent 5-azacytidine (5-aza) and HDAC inhibitor Trichostatin A (TSA) in combination, indicating that these polyphenols can be used as a chemotherapeutic strategy for breast cancer [119]. The combination was shown to reduce the gene expression of HDAC1 in the mRNA level in the MDA-MB-231 cells and decrease HDAC1 protein level in MDA-MB-157 cells. The combination can affect DNMT expression in breast cancer as well [118].

The combinatorial effects of the polyphenols were also observed in vivo through analysis of mouse xenografts [118]. The combination treatment significantly decreased tumor growth compared to individual treatment. The dietary combination exhibited similar results as the in vitro results by reactivating ERα expression and reducing the expression of HDAC1 and DNMT1. Chromatin immunoprecipitation (ChIP) assays showed that the combinatorial treatment increased the histone acetylation chromatin activators acetyl-H3, acetyl-H3K9 and acetyl-H4. This effect was amplified when the mice were treated with the polyphenol combination along with tamoxifen, an anti-estrogen chemotherapeutic agent. Finally, this combination treatment was able to decrease the binding of the transcriptional co-repressor SUV39H and increase the binding of the transcriptional co-activator, P300 [118]. A conflicting result from this study is that 5-aza and TSA have shown dangerous side effects, which could limit the combination’s translational applicability. In addition, there is a variability in responsiveness among different breast cancer cell lines, indicating that the results need further validation. However, the usage of EGCG and SFN are found in common dietary items (green tea and broccoli sprouts), meaning that it has significant translational potential. In addition, this study looked at the combination effects in both in vitro and in vivo models.

The combinatorial effects of these polyphenols were studied on breast cancer as well [120]. It was demonstrated that the combination treatment caused increased apoptosis in breast cancer cells in comparison to normal cells while still maintaining decreased toxic effects on normal cells. Flow cytometry results indicated that EGCG and SFN increased the number of cells in the S-phase inducing cell cycle arrest. The investigation also studied genome-wide DNA methylation alterations, and the results show that there was a significant difference in the DNA methylation status between the control, individual treatment of each polyphenol, and the combination treatment. Many genes that had altered methylation led to breast cancer suppression. After functional gene ontology analysis, it was revealed that the combination affects chromosomal structure and RNA binding facilitates anti-cancerous effects. The combination also led to an upregulation of the tumor suppressor gene *DCBLD2* and downregulation of the tumor-promoting gene *Septin 9* [120]. Further investigation of the combinatorial effects of both polyphenols in breast cancer showed a repression of gene expression of *DNMT3A*, *HDAC6*, *KAT2A*, and *EZH2* [121]. Through administration of EGCG and SFN as a diet for mice, it was concluded that the polyphenols had no effect on growth performance for the HER2/neu and CS (1)-SV40 Tag (C3) offspring mice, prevented ER- mammary tumorigenesis in C3 and HER2/neu mice, and increased the expression of tumor suppressors and decreased the expression of tumor-promoting proteins for C3 mice. Specifically, it increased the expression of P16, P53, and myc and decreased the expression of HDAC1, HDAC3, and HDAC8. The SFN and EGCG combination was also tested to determine if their synergism can have transgenerational effects. After harvesting sperm samples from C3 mice and performing KEGG and GO pathway analyses, it was concluded that the combination affects cell adhesion molecules, mTOR signaling pathways, NF-kappa B signaling pathways, and Ras protein signal transduction. This shows that the combination regulates pathways responsible for cancer progression and metastasis in future generations. Reduced representation bisulfite sequencing (RRBS) analysis of the mice sperm showed that there was no significant difference in methylation patterns in mice sperm for mice that received the combination treatment compared to the control group [121]. Utilizing mice studies provides many benefits, but there are various factors that can affect and change the results of these models. For example, the diet can impact the outcomes of animal studies, which may limit the clinical impact of their results.

#### 5.3.2. EGCG and Cytokine TRAIL Receptors

Cytokine TRAIL targets TRAIL receptors and induces tumor regression while having minimal side effects [122]. There have been experiments examining the synergistic effects of EGCG and TRAIL. Hepatocellular carcinoma cells were treated with TRAIL and EGCG. The combination enhanced the cytotoxicity compared to cells that were treated with the compounds individually. In addition, the co-treatment had a synergistic effect in downregulating the protein expressions of Bcl-2α and Bcl-xl [123].

Evaluation of the synergistic effects of both compounds for pancreatic cancer has been conducted [64]. TRAIL can interact with death receptors to induce apoptosis. The combination treatment enhanced PARP cleavage, which leads to increased apoptosis. Through clonogenic survival assays, it was shown that there was decreased colony growth in the combination treatment compared to treatment with the individual compounds [124].

An additional investigation observed the combination in renal cell carcinoma cells [125]. Through cytometry analysis, it was indicated that the combination significantly increased apoptotic cell population compared to individual treatment. There was also an increased loss of viability in cells as well as elevated activities of the caspase-3, caspase-8, and caspase-9 proteins. The combination had decreased the levels of Bcl-2, Mcl-1, and c-FLIP, which leads to increased apoptosis. Specifically, the mRNA levels of these molecules significantly decreased. The RT-PCR results revealed the increased activities of the caspase-3, -8, and -9 proteins [125]. This study demonstrates multiple ways the combination leads to apoptosis by exploring various caspase pathways as well as pro and anti apoptotic protein activity levels. Another strength of the study is that it examines cancers that are resistant to TRAIL-based therapies to see how it can overcome this resistance. This further underscores the clinical impact of the study.

#### 5.3.3. EGCG and Cyclooxgenase-2 (COX-2)

COX-2 inhibitors can be used for cancer chemoprevention [126]. NS-398 is a type of COX-2 inhibitor, and this molecule was used in combination with EGCG as a treatment for prostate cancer cells [127]. MTT assays showed that the combination increased the cell growth inhibition by 15–28% compared to individual treatment with each agent. The combination also led to increased apoptosis, as there was increased protein expression of Bax and decreased protein expression of Bcl-2. Immunoblot analysis showed that the combination increased the protein expressions of caspases, which leads to increased apoptosis. There was inhibition of the cancer-progressing pathways NF-kB and PPAR-y. In vivo studies with mice showed an 81% inhibition of tumor growth with the combination treatment compared to 42% inhibition with EGCG alone and 57% with celecoxib alone. There was also a decrease in prostate-specific antigen (PSA) levels, which correlates to decreased levels of IGF-I, which leads to increased cell proliferation. Similar to the in vitro results, there was an increase in Bax levels and a decrease in Bcl-2 levels in mice, indicating a trend towards apoptosis [127]. While this study demonstrates both in vitro and in vivo studies, there are no studies with humans. Showing experiments involving clinical trials can enhance the medical impact of this combination and whether it can be used as an effective treatment for cancer.

#### 5.3.4. EGCG and Clofarabine (CIF)

CIF can regulate many epigenetic processes exerting anti-cancer effects [128]. MTT assays demonstrated that the combination of EGCG and CIF increased the inhibition of cell growth in MCF7 and MDA-MB-231 breast cancer cells compared to CIF by itself. However, there was not a significant change in cell death when EGCG was used in combination with CIF. In addition, the combination did not cause significant cell necrosis, as indicated by the flow cytometry. RARB promoter is responsible for cell growth, differentiation, and apoptosis. When it becomes hypermethylated, it can silence its expression, causing the development of cancer [129]. The combination of CIF with EGCG led to an increased inhibitor effect of RARB promoter methylation. There was also a 20-fold increase in RARB mRNA level. These effects were even more prominent when the combination was used along genistein as well. The CIF and EGCG combination also revealed an increased CDKN1A expression compared to CIF alone. In addition, this combination also enhanced DNMT1 downregulation by 14% compared to CIF alone. Finally, the combination led to decreased PTEN methylation, which leads to increased expression of the PTEN gene, which suppresses the formation of cancer [130].

#### 5.3.5. EGCG and Sodium Butyrate

Sodium Butyrate (NaB) is a dietary microbial fermentation product of fiber and is used as an overexpressed anti-apoptotic protein in colon cancer cells [131]. This study observed the effects of EGCG and NaB in colorectal cancer. The effect of both compounds presented an additive effect, as there was higher inhibitory concentration (IC) values indicating an increased inhibition from the combination [132]. Morphological changes were also tested, and it was observed that the treatment group with the combination had a flattened, circular characteristic demonstrating normal epithelial-like morphology. This contrasted with the control group that did not receive the treatment and had a more round morphology. The combination induced apoptosis and induced cell cycle arrest, particularly in the G2/M phase. Through colonogenic analysis, the combination was shown to inhibit colony formation by 80%, while EGCG by itself inhibited colony formation by 20% and NaB inhibited the colonies by 50%. PCR analysis indicated that p21 levels increased significantly for the combination, which is responsible for DNA repair processes. In addition, the combination also led to a decrease in DNMT1 levels and HDAC1 levels. This was due to the fact that EGCG inhibits the catalytic site of DNMT1, and NaB can also decrease DNMT1 levels in breast and prostate cancer [133,134]. Survivin is a protein that is often overexpressed in colorectal cancer, which leads to the inhibition of apoptosis, cell proliferation, and tumor metastasis [135]. The combination decreases the expression of this protein, which inhibits the formation of colorectal cancer. EGCG and NaB were shown to activate p53, NF-kB-p55 and y-H2Ax H3, as well as inhibit DNMT3A and DNMT3B [132].

#### 5.3.6. EGCG and Epicatechin

Epicatechin (EC) is another type of polyphenol similar to EGCG except it does not have a galloyl moiety [136]. The combination of these green tea polyphenols have been studied as a treatment option for cancer. One study evaluated the combination for human lung cancer. The results show that the combination induced DNA fragmentation and apoptosis in the PC-9 lung cancer cell line [137]. In addition, the combination created cell growth inhibition, as they led cells to have cell cycle arrest in the G2-M phase. For instance, after 2 days with the cells being treated with EGCG alone, the number of cells was at 73.3%. For EC alone, the cells were at 97.8%. However, when both compounds were used in combination, the number decreased to 27.8%, signifying increased growth inhibition. TNF-α is an endogenous tumor promoter and has been shown to promote carcinogenesis in lungs. It was shown that utilizing EC enhances the inhibition of TNF-α release when used in combination with EGCG [137].

The combination of these compounds has also been evaluated in gastric cancer. The compounds were shown to have a synergistic effect on growth inhibition and apoptosis. EC by itself had no effect on the growth inhibition, but when used in combination with EGCG, there was increased growth inhibition and induction of apoptosis. Specifically, the activity levels of caspse-3, caspase-8, and caspase-9 were increased [132,138].

#### 5.3.7. EGCG and Soy Products

Soy products have been shown to reduce prostate cancer in men [139]. Thus, the combinatorial effects of soy products and EGCG have been studied. The combination of soy phytochemicals and tea in vivo were studied as part of a mouse model. Mice that were fed the combination had a lower rate of tumorigenicity [140]. The combination was shown to inhibit tumor metastasis in lymph nodes. When each compound was used by itself, it did not significantly reduce the tumor metastasis of lymph nodes; however, when used in combination the reduction was significant. The tumor weight was reduced as well. When green tea was given by itself, it reduced the tumor weight by 22%. When the combination was used, the tumor was reduced by 88%. PSA levels were also decreased when the combination was used. Finally, proliferation of primary tumors was decreased and dihydrotestosterone (DHT) levels were lowered [140].

Another group performed an in vivo study in mice of the combinatorial effects of soy phytochemicals and tea in human breast cancer [141]. The combination was shown to reduce the tumor weight by 56%, while the combinations by themselves had little effect on tumor size or tumor weight. There were no synergistic effects of the soy and green tea on tumor proliferation and tumor apoptotic index; however, there were significant effects on the inhibition of tumor angiogenesis. Soy and green tea by themselves did not have a significant effect on the expression of ER-α, but the combination significantly inhibited this expression in vivo. The combination was also able to reduce the serum levels of IGF-I [141].

#### 5.3.8. EGCG and Cisplatin

Cisplatin (DDP) is a drug treatment for non-small-cell lung cancer. DDP causes DNA interstrand and intrastrand crosslinks, which leads to an inhibition of cell replication and transcription [142]. Researchers in one study exposed one cell line of non-small-cell lung cancer, A549, to DDP [143]. They then treated this cell line with EGCG and DDP, and it was shown that the combination inhibited cell growth, caused G1 phase cell cycle arrest, and increased apoptosis. In addition, the compounds had a synergistic effect in decreasing the activity of DNMT and HDAC. The combination also restored the expression of the genes GAS1, responsible for inhibiting cell proliferation, TIMP4, inhibitor for MMPs, ICAM1, which plays a role in immune responses and inflammatory processes, and *WISP1*, a gene important for tissue regeneration and development. In vivo studies in animal models demonstrated the synergistic effects of the compounds, as there was tumor reduction and decreased methylation of genes *GAS1*, *TIMP4*, *ICAM1*, and *WISP1* [143]. Longer-term studies could be incorporated to determine the chronic effects of the combination and to observe whether the same anti-cancerous effects occur over longer periods of time.

#### 5.3.9. EGCG and Rapamycin and Indocyanine Green

Rapamycin (RAPA) is an immunosuppressive agent that has been shown to suppress tumor growth and proliferation [144]. A study observed the effects of combining RAPA, EGCG, and indocyanine green (ICG) nanoparticles creating IRE NPs [145]. After combination, these IRE NPs had higher toxicity in the MCF-7, HepG2, and HeLA cells compared to the compounds administered singly. In vivo studies showed inhibition of tumor growth in the H22 tumor-bearing mice while maintaining minimal side effects. IRE NPs were also combined with laser irradiation to determine if there would be a synergistic effect. This combination indeed demonstrated a synergistic effect, as it enhanced cell death in all the cell lines with the IC50 value decreasing. For instance, the IC50 value for just the IRE NPs alone for the MCF-7 cells was 49.36 uM, while the IC50 value for the IRE NPs with the laser irradiation towards MCF-7 was 14.52 uM. Apoptotic effects were observed through flow cytometry, which showed that the IRE NPs with or without laser led to reduced cell viability. This is in contrast to ICG used alone, which did not have a significant effect on HepG2 cells. These results highlight the combinatorial effects of rapamycin with EGCG [145]. This study shows a variety of results that show how the combination can have a suppressive effect on in vitro cell lines. However, there are no studies using in vivo models. Further studies in vivo can show more about the clinical potential of the combination and its effects on tumor growth and size.

#### 5.3.10. EGCG and Luteolin

Luteolin is another natural dietary polyphenol that has been used for cancer prevention [146]. This polyphenol was studied alongside EGCG to see if there was any synergism directed towards head and neck cancer as well as lung cancer. The combination was first used on the Tu212 SCCHN cell line, and the results indicated that the combination enhanced apoptosis. The combination was also shown to affect various apoptosis pathways, such as increased cleavage of caspase 8 and 3, upregulation of DR5, and decrease in tBID. Specifically, luteolin and EGCG used together created 3–5 times more apoptosis than when used separately, as well as more efficient PARP cleavage. Colony formation assays showed that the combination increased inhibition of colony growth more than usage of each agent alone. Luteolin and EGCG also were able to stabilize and promote phosphorylation of the p53 protein at Ser15, allowing it to become active and localize in the mitochondria and promote apoptosis. The combination also induced the expression of γ-H2AX, which increases DNA double-strand breaks. In vivo mice studies showed that the combination increased tumor inhibition while maintaining stable body weights, which limited organ toxicity [147].

A summary of the combinatorial effects of EGCG with other polyphenols is provided in Table 5 and Table 6 below.

## 6. Other Combinations of Compounds

Table 7 below shows further combinations of different polyphenols, the genes they affect, the specific anti-cancerous effect they exhibit, the in vitro cell line and/or the in vivo model the experiment was conducted using, and the appropriate references. The most common anti-cancerous effects exhibited were PARP cleavage, increase in c caspase-3 levels, increased cytotoxicity, increased cell population in the G2/M phase, and ROS accumulation.

## 7. Addressing Clinical Aspects of Polyphenols

Many of these polyphenols have significant anti-cancer effects but are limited by their bioavailability. One way to overcome this is to create protein-based nanoformulations. For instance, using protein-based nanoformulations creates self-assembled micelles that can improve the solubility and antioxidant activity of polyphenols. Specially, using casein has been shown to enhance curcumin’s therapeutic effects against cancer [163]. In addition, gelatin can encapsulate polyphenols like EGCG and resveratrol to increase its efficacy. There are also polysaccharide-based nanoformulations that use chitosan, which can improve the absorption of polyphenols and prolong their effects. Furthermore, chitosan has the ability to interact with various functional groups, allowing it to be versatile material for targeted delivery. Similar to polysaccharide nanoformulations, lipid-based nanoformulations can also lead to prolonged drug release and bioadhesion [163]. Phytosome delivery systems can also increase the bioavailability of polyphenols by enhancing the rate and solubilization of polyphenols, which allow it better cross cell membranes [164].

While the majority of the studies for polyphenols are conducted in vitro, there are a few studies that highlight the therapeutic effects of polyphenols in clinical trials. One study observed the effects of curcumin with 5-FU or oxaliplatin in colorectal cancer. These clinical trials showed that curcumin was a safe treatment for patients with advanced colorectal cancer. In addition, when used with 5-FU or oxaliplatin, curcumin enhanced its anti-tumor effects and reduced cancer stem cells in colorectal liver metastases. However, there are still some concerns with the usage of curcumin for humans, including its limited bioavailability and rapid metabolism. In addition, in high doses curcumin can cause hepatotoxicity and interfere with iron metabolism [165].

EGCG has also been shown to enhance cancer chemotherapy. Specially, it can increase the efficacy of the chemotherapeutic drugs cisplatin, 5-FU, doxorubicin, and tamoxifen. It is able to reduce the resistance of cancer stem cells to chemotherapeutic treatment as well as enhance drug efficacy by modulating uptake transporters. It is able to also induce apoptosis and cell cycle arrest through the p53, caspase, and mitochondria pathways. However, similar to curcumin, EGCG also has low bioavailability and side effects at high concentrations. In addition, it is poorly absorbed orally and can sometimes be antagonistic with chemotherapeutic drugs [166].

## 8. Conclusions

Cancer is one of the leading causes of death worldwide, which creates a pressing need to develop better and more effective treatments. There are many existing treatments of cancer, including chemotherapy, small-molecule drugs, monoclonal antibody therapy, and gene therapy that have been shown to be successful by regulating the epigenetic mechanisms of cancer. However, several studies demonstrating the synergism of combination treatment with polyphenols demonstrates an alternative preventive treatment for cancer that is both effective and safe. Specifically, there have been numerous studies performed in vitro showing how different combinations at the appropriate concentrations can lead to an inhibition of cancer progression in cell lines, increased apoptosis, usage as DNMT and HDAC inhibitors, and inflammatory stress. While there are fewer in vivo studies that have been conducted, those that have been reported demonstrate a decrease in tumor growth and cancer metastasis, supporting the idea that combinatorial treatment using polyphenols is a promising preventive option for cancer. Further studies should be conducted in vivo, including animal models and clinical trials, to provide more support and evidence for this treatment. In addition, more studies on new combinations of polyphenols can also be performed, as there are thousands of polyphenols that have medicinal potential, making the different possibilities of combination limitless. Furthermore, a limitation of this review article is that it does not adequately address the combinatorial effects of polyphenols in human clinical trials. A deeper exploration of how these polyphenols have been studied in clinical trials would provide more insight into the therapeutic efficacy of combinatorial treatment.

## Figures and Tables

**Table 1:** Summary of preclinical studies of curcumin in combination with other molecules.

Compounds	Cancer	Anti-Cancer Effects	References
Curcumin and EGCG	Colorectal Carcinoma	Blocks JAK-STAT3 signaling mRNA and protein levels of TEC markers and IL-8 were reduced	[64]
	Prostate cancer	p21 increased Inhibition of *p*-Rb	[65]
Curcumin and Resveratrol	Colorectal cancer	Increase in apoptosis by 300% Cell cycle arrest in G2-M phase Decrease in gene expression for PMAIP1, BID, ZMAT3, CASP3, and FAS 50% inhibition of tumor growth in SCID mice	[66]
Curcumin, Resveratrol, and benzopyrene	Prostate cancer	Increase p21 levels Maintain zinc levels	[68,69]
Curcumin and Doxorubicin	Breast cancer	Increased cytotoxicity	[72]
	Leukemia and lymphoma	mRNA expression of MDR1 and Bcl-2 in CML Reduced drug resistance of doxorubicin	[74]
Curcumin and Paclitaxel	Cervical cancer	Downregulation of NF-kB activation Apoptosis and G2/M cell cycle arrest Upregulation of surviving and Bcl-2 protein	[77]

**Table 2:** Summary of clinical studies of curcumin in combination with other molecules.

Compounds	Cancer	Anti-Cancer Effects	References
Curcumin and Resveratrol	Colorectal cancer	Increase in apoptosis by 300% Cell cycle arrest in G2-M phase Decrease in gene expression for PMAIP1, BID, ZMAT3, CASP3, and FAS 50% inhibition of tumor growth in SCID mice	[66]
Curcumin and Tocotrienol-rich fraction Vitamin E	Colon cancer	Increased number of microbial bacterial species in SCID mice Richness and diversity of bacterial species increased by 44% in SCID mice	[70]
Curcumin, 5-flurouracil and oxaliplatin	Colorectal cancer	Decreased growth of primary cancer stem cells by 80% for patients with colorectal liver metastases Decreased pluripotent stem cell markers such as Oct3-4, AFP, and HNF/FOxAe	[71]

**Table 3:** Summary of preclinical studies of resveratrol in combination with other molecules.

Compound	Cancer	Anti-Cancer Effects	References
Resveratrol and piperine	N/A	Inhibits metabolism of resveratrol prolonging its effects	[78]
Resveratrol and quercetin	Skin cancer	Decrease oxidative stress and inflammation Inhibited migration of cells	[79]
	Glioma cancer	Decrease cells by 80% Activation of caspase 3/7 activity	[80]
	Neointimal hyperplasia	Decreasing serum amyloid A and soluble vascular cell adhesion molecules	[82]
Resveratrol, quercetin, and ellagic acid	Leukemia	Decreased cell viability Increased caspase 3 activity	[81]
Resveratrol and pterostilbene	ERα- negative breast cancer	Increase in ERα protein expression Decrease in acetyl-H3 and acetyl-H4 histone markers Decrease in DNMT and HDAC activity and increase in HAT activity	[83]
	Breast cancer	Cell cycle arrest in G2/M and S phase Decrease in Phospho-H2AX levels Downregulation of human telomerase reverse transcriptase	[84]
Resveratrol and arsenic trioxide	Human lung adenocarcinoma and hepatocellular carcinoma cells	Increases Nrf2 activation ROS accumulation and increase in ER stress related proteins Increased caspase 3 activation	[86]
Resveratrol and grape seed proanthocyanidins	Breast cancer	Decrease in cell viability Reduction in colony formation Increase in apoptosis and decrease in cell density Upregulation of Bax protein and downregulation of bcl-2 Decrease in DNMT and HDAC activity	[88]
Resveratrol, EGCG, and γ-tocotrienol	Breast cancer	Decrease in cell number Inhibition of colony formation Increase in Bcl-2/Bax ratio Not much change in activities of antioxidant enzymes	[89]
Resveratrol and oxaliplatin	Colorectal cancer	Decrease in cytotoxicity Decrease in tumor weight in mice Decrease expression of α-SMA and CCGBP1 Reduction in myeloid-derived suppressor cell	[92]
Resveratrol and 5-flurouracil (5-FU)	Skin cancer	Increase apoptosis and intervene DNA synthesis Increase expression of p53 Improved antioxidant activity Downregulation of NF-kB activity and inhibition of phosphorylation of STAT-3	[95]
	Skin cancer	Greater delivery of drugs and prolonged release of drugs	[96]
Resveratrol and Endoplasmic reticulum (ER) stress activators	Gastric tumors	Decreased levels of migration proteins MMp2 and MMp9 Increased ER stress proteins	[97]
Resveratrol and tamoxifen	Breast cancer	Inhibition of breast cancer cells 70–80% inhibition of proliferation Increase in late apoptosis Increased expression of beclin-1 Increased expression of ERα and c-Myc	[100]
Resveratrol and cytochalasin D	colorectal cancer	Decrease expression of Sirt1 protein Increased apoptosis and caspase-3 expression Greater invasion-inhibition ability Suppression of integrin expression and NF-kB	[104]
Resveratrol and salinomycin	breast cancer	Decreased cell viability Downregulation of proteins in Wnt signaling Downregulation of CDK2 and CDK4 proteins Decrease expression of PARP, caspase-8, and caspase-9	[107]
Resveratrol and pemetrexed	Non-small cell lung cancer	Decreased ERCC1 mRNA expression and stability Enhanced luciferase activity	[109]
	Lung cancer	Increased cytotoxic effects Reduction in lung tumor weight in mic Fewer malignant lesions	[109,110]
Resveratrol and clofarabine and all-trans retinoic acid	Chronic myeloid leukemia	Decrease in DNMT1 expression Enhanced CDKN1A protein levels 60–70% increase in RARB expression Increase in apoptosis and caspase-3 activation	[113]

**Table 4:** Summary of clinical studies of resveratrol in combination with other molecules.

Compound	Cancer	Anti-Cancer Effects	References
Resveratrol and melatonin	Breast cancer	Decrease tumor incidence by 17% in rats Decrease in number of invasive carcinomas	[87]
Resveratrol and oxaliplatin	Colorectal cancer	Decrease in cytotoxicity Decrease in tumor weight in mice Decrease expression of α-SMA and CCGBP1 Reduction in myeloid-derived suppressor cell	[92]
Resveratrol and 5-flurouracil (5-FU)	Liver cancer	Greater inhibition of cancer cells in mice with liver cancer Induced S phase arrest	[94]
Resveratrol and pemetrexed	Lung cancer	Increased cytotoxic effects Reduction in lung tumor weight in mic Fewer malignant lesions	[109,110]

**Table 5:** Summary of preclinical studies of EGCG in combination with other molecules.

Compound	Cancer	Anti-Cancer Effects	References
EGCG and sulforaphane	Breast cancer	Increased ERα re-expression in ER-negative breast cancer cells Reduced gene expression of HDAC1 Decreased tumor growth in mouse xenografts Increased histone acetylation chromatin activators Decrease the binding of the transcriptional co-repressor SUV39H and increase the binding of the transcriptional co-activator, P300	[118]
	Breast cancer	Increased S phase cell cycle arrest Alteration of DNA methylation Upregulation of the tumor suppressor gene DCBLD2 and downregulation of the tumor-promoting gene Septin 9 Increased tumor suppressors and decreased expression of tumor-promoting genes Affects cell adhesion molecules, mTOR signaling pathways, NF-kappa B signaling pathways, and Ras protein signal transduction	[121]
EGCG and cytokine TRAIL receptors	Hepatocellular carcinoma cells	Downregulating the protein expressions of Bcl-2α and Bcl-xl	[123]
	Pancreatic cancer	Increased PARP cleavage Decrease in colony growth	[124]
	Renal cell carcinoma cells	Increased apoptosis Elevation of caspase-3, caspase-8, and caspase-9 proteins Decreased the levels of Bcl-2, Mcl-1, and c-FLIP	[125]
EGCG and cyclooxgenase-2 (COX-2)	Prostate cancer	Cell growth inhibition by 15–28% Increased protein expression of Bax and decreased protein expression of Bcl-2 Inhibition of NF-kB pathway and PPAR-y pathways Decreased prostate-specific antigen levels	[127]
EGCG and clofarabine (CIF)	Breast cancer	Cell necrosis Increased inhibitory effect of RARB promoter methylation Increased CDKN1A expression Decreased PTEN methylation	[130]
EGCG and sodium butyrate	Colorectal cancer	Flattened, circular cells Increased apoptosis and cell cycle arrest in G2/M phase Inhibition of colony formation by 80% p21 levels increased Decreased DNMT1 and HDAC1 levels Inhibition of survivin	[132]
EGCG and epicatechin	Lung cancer	DNA fragmentation Cell cycle arrest in G2/M phase and apoptosis Inhibition of TNF-α release	[137]
	Gastric cancer	Increase activity levels of caspse-3, caspase-8, and caspase-9	[132,138]
EGCG and cisplatin	Non-small cell lung cancer	Increased G1 phase cell cycle arrest Decrease activity of DNMT and HDAC Restored expression of GAS1, TIMP4, ICAM1, and WISP1	[143]
EGCG and rapamycin	Breast, liver, and cervical cancer	Increased toxicity Decreased IC50 value	[145]
EGCG and luteolin	Head and neck cancer and lung cancer	Increase cleave of caspase 8 and 3 Upregulation of DR5 Decrease in tBID Increased inhbition of colony growth Increased phosphorylation of p53 protein Increased expression of γ-H2AX	[147]

**Table 6:** Summary of clinical studies of EGCG in combination with other molecules.

Compound	Cancer	Anti-Cancer Effects	References
EGCG and soy products	Prostate cancer	Lower rate of tumorigenicity in mice Inhibition of tumor metastasis in lymph nodes Reduction in tumor weight and PSA levels	[140]
	Breast cancer	Reduce tumor weight by 56% Inhibition of tumor angiogenesis Reduce serum levels of IGF-I	[141]

**Table 7:** Combinations of different polyphenols.

Compounds	Gene Target	Anti-Cancer Effects	In Vitro Cell Lines	In Vivo Model	References
Oridonin and wogonin	*p53*, *bcl*, *bax*, *Akt*, *PARP*, *caspase 3*	Inhibition of colony formation Modulation of cell cycle Increased apoptosis PARP cleavage *p53* stabilization No change in Bcl-2/Bax protein expression Akt1 increased expression	A2780 and PTX10 ovarian cell lines	N/A	[148]
Selenium and tocopherol	N/A	Reduced total mortality and total cancer mortality Reduced gastric cancer incidence Reduced levels of oxidative DNA damage	N/A	Clinical trials with prostate cancer patients	[149]
Calcitrol and dexamethasone	N/A	Increased vitamin D receptor ligand binding in the tumor -decreased tumor growth	Prostate cancer cell lines	Clinical trials with prostate cancer patients	[150,151]
Pomegranate juice polyphenols	N/A	Suppression of cancer cells by 70%	DU 145 human prostate cancer cells	N/A	[136]
Muscadine grape skin extract and trastuzumab	*p27* *HER2*	Reduced activation of protein kinase B (AKT) pathway Reduced HER2 protein expression Increased p27 levels and FOXO1 Inhibited proliferation	TRZ-sensitive SKBR3 and -resistant HCC1954 human HER2 breast cancer cells	N/A	[152]
Sulforaphane and diindolylmethane	N/A	G2/M cell cycle arrest Cell growth inhibition Increased PARP cleavage	40-16 colon carcinoma cells.	N/A	[153]
Cisplatin and honokiol	*ACOX1* *CPT1* *p-HSL* *p-PLIN* *Xrcc1* *caspase-3*	Increased ROS production Reduction in DCF signals Decreased sperm motility Reduced ER stress in Testes Increased apoptosis through upregulated caspase-3 Elevation of the lipid/fatty acid oxidation related genes: *ACOX1*, *CPT1*, *p-HSL*, and *p-PLIN*	Mouse Sertoli cells	N/A	[154]
Honokiol and pemetrexed	*VEGF-1* *Caspase-3* *Ki-67*	Increased cytotoxicity Enhanced receptor mediated endocytosis Reduced tumor volume Increased caspase 3 expression levels Decreased expression of VEGF-1 Downregulation of the Ki-67 protein	MDA-MB-231 and A549	Mice	[155]
Doxorubicin and FeNCP	N/A	Increased ROS levels	HepG2, B16F0, and Vero cells	N/A	[156]
TRAIL and hydroxychavicol	*XIAP* *FLIP*	Increased cytotoxicity Enhanced caspase-3, -8, and PARP cleavage Reduction in antiapoptotic proteins Increase intracellular ROS levels	K562 CML cells	N/A	[157]
Piceatannol and everolimus	*Beclin-1* *LC3B* *Ki67* *mTOR* *PDK* *PIK3CA* *ULK1* *Bcl-2*	Inhibition of cell proliferation and colony formation Increased autophagy and apoptosis Decreased expression of mTOR, Bcl-2, PDK1, PIK3CA, and ULK1 genes Inhibition of tumor progression Increased Beclin-1 activity	Gastric cancer cells SGC7901	Mice model	[158]
DNA methyltransferase (DNMT) inhibitor 5-aza-dC with ionizing radiation (IR)	*DNMT1* *p21* *p15*	Reduction in tumor cells Decreased cell viability Increased DNA damage response Increase levels of *p53* and *p21* DNA double strand breaks	Medulloblastoma cells D283-Med, MEB-Med8a, and DAOY	Mouse brain slice culture model	[159,160]
Anthocyanin rich berry extracts and SN-38	*NF-kB* reporter genes	Prevented cytotoxic effects of SN-38 Increased DNA double strand breaks Reduced DNA/topoisomerase1 covalent complexes Reduce cell viability Anti-oxidative properities Reduced expression of NF-kB protein	Human epithelial cells HCEC-1CT	N/A	[145]
Lasota strain of newcastle disease virus and oleurpein (oil leaf extract)	*PARP* *Caspse-3*	Increased cytotoxicity Increased early apoptosis *PARP* cleavage *Caspse-3* activation	HeLa and HDF cervical cell lines	N/A	[161]
Gemcitabine and F1	*p53* *Bcl-2* *Bak* *Bax* *PARP* *Procaspase-3* *Caspase-3*	Increase cell population in the G2/M phase Decrease in cells in the G0/G1 phase Increased cell cycle arrest ROS generation Increased phosphorylation of ATM, Chk-1, and Chk-2 Reduction in cdc25c levels	PaCa-2 pancreatic cancer cells	N/A	[162]
Rottlerin and camptothecin (topoisomerase 1 inhibitor)	*P21* *Bcl* *bad*, *bax*	DNA fragmentation Stabilized topoisomerase 1-DNA complexes Increased mitochondrial stress Increased cleavage of caspase-3, PARP-1, PKC δ	PC-3 HRPC prostate cancer cells	N/A	[145]

N/A for In Vivo Model indicates that the study did not conduct an experiment with In Vivo Models.

## Abbreviations

5-FU	5-Fluorouracil
AMPK	Adenosine monophosphate-activated protein kinase
CA	Calcium
CaMKII	Calcium/calmodulin-dependent protein kinase II
CDK	Cyclin-dependent kinases
CML	Chronic myeloid leukemia
COX	Cyclooxygenase
DNMT	DNA methyltransferase
E2F	Early region 2 binding factor
EMT	Epithelial–mesenchymal transition
ER	Endoplasmic reticulum
Erα	Estrogen receptor alpha
GO	Gene ontology
HAT	Histone acetyltransferases
HDAC	Histone deacetylase
IGF-I	Insulin-like growth factor 1
IL-8	Interleukin-8
IκB	Inhibitor of nuclear factor kappa B
JAK	Janus kinase
KEGG	Kyoto Encyclopedia of Genes and Genomes
MAPKs	Mitogen-activated protein kinases
miRNA	microRNA
mTOR	Mammalian target of rapamycin
NQO1	H quinone dehydrogenase 1
NRF2	Nuclear factor erythroid 2-related factor 2
PARP	Poly ADP-ribose polymerase
*p*-Rb	Retinoblastoma protein
PI3K/AKT	Phosphoinositide 3-kinases
ROS	Reactive oxygen species
RT-PCR	Reverse transcription polymerase chain reaction
SCID	Severe combined immunodeficiency
SOD	Superoxide dismutase
STAT3	Signal transducer and activator of transcription 3
TEC	Thymic epithelial cells
